# Semantic Segmentation of Medical Images Based on Runge–Kutta Methods

**DOI:** 10.3390/bioengineering10050506

**Published:** 2023-04-23

**Authors:** Mai Zhu, Chong Fu, Xingwei Wang

**Affiliations:** 1 School of Computer Science and Engineering, Northeastern University, Shenyang 110819, China; zhumai@stumail.neu.edu.cn (M.Z.); wangxw@mail.neu.edu.cn (X.W.); 2 Engineering Research Center of Security Technology of Complex Network System, Ministry of Education, Shenyang 110819, China; 3 Key Laboratory of Intelligent Computing in Medical Image, Ministry of Education, Northeastern University, Shenyang 110819, China

**Keywords:** semantic segmentation, convolutional neural network, dynamical system, Runge–Kutta methods

## Abstract

In recent years, deep learning has achieved good results in the semantic segmentation of medical images. A typical architecture for segmentation networks is an encoder–decoder structure. However, the design of the segmentation networks is fragmented and lacks a mathematical explanation. Consequently, segmentation networks are inefficient and less generalizable across different organs. To solve these problems, we reconstructed the segmentation network based on mathematical methods. We introduced the dynamical systems view into semantic segmentation and proposed a novel segmentation network based on Runge–Kutta methods, referred to hereafter as the Runge–Kutta segmentation network (RKSeg). RKSegs were evaluated on ten organ image datasets from the Medical Segmentation Decathlon. The experimental results show that RKSegs far outperform other segmentation networks. RKSegs use few parameters and short inference time, yet they can achieve competitive or even better segmentation results compared to other models. RKSegs pioneer a new architectural design pattern for segmentation networks.

## 1. Introduction

Deep learning has recently achieved success in many fields [1,2,3,4,5,6]. In particular, deep convolutional neural networks have greatly advanced the progress of medical image segmentation. The state-of-the-art segmentation networks are typically encoder–decoder structures. Network models for image classification are usually adopted as the backbones of semantic segmentation networks [7,8,9,10]. The backbone is also referred to as the encoder. Correspondingly, there is a decoder following it. Skip connections closely connect the encoder and decoder. Consequently, the design of the segmentation network is fragmented and lacks overall consideration. Furthermore, the design lacks a mathematical explanation. As a result, there are several issues in medical image segmentation networks. First, segmentation networks are very large and computationally expensive. Second, the performance of segmentation networks often varies across different organs, and the generalizability is poor. Therefore, it is necessary to redesign the segmentation network with overall consideration and mathematical explanation.

From experience in image classification [11,12,13,14,15,16], the dynamical systems view is a good perspective for designing efficient network models with appropriate mathematical interpretation. Reference [11] views the forward pass of a neural network as the trajectory of a dynamical system described by an ordinary differential equation (ODE). Since the trajectories of dynamical systems are usually approximated by numerical methods, numerical methods are also used to construct neural networks. The Runge–Kutta (RK) methods are frequently used numerical methods [17]. They are used to construct Runge–Kutta convolutional neural networks (RKCNNs) [16]. RKCNNs are state-of-the-art numerical networks for image classification. They are very efficient and save computing resources significantly. They also surpass the models using linear multi-step methods, another kind of numerical method.

No segmentation model has been constructed from the dynamical systems view, although semantic segmentation is based on image classification. To obtain higher segmentation efficiency, the construction of a numerical segmentation network is a valuable research topic.

We abandoned the concept of encoder and decoder in segmentation models and instead model the entire network from the dynamical systems perspective. We regarded the process of semantic segmentation as a dynamical system since it is also the neural network’s forward pass, just like the image classification. Similarly, we used the RK methods in the segmentation networks to approximate the trajectory of the dynamical system. Due to the superiority of RKCNN, we exclusively used RKCNN as a reference in order to construct segmentation networks.

Unlike all of the existing numerical models including RKCNNs, we creatively used multiple scales within one time step of RK methods. In other words, the existing models maintain the same scale within a time step, while we down-sampled and up-sampled within a time step. Different stages approximated the increment of the time step in different scales. Consequently, we proposed a novel segmentation network structure using the RK methods. It is called RKSeg. Moreover, we evaluated the performance of RKSegs on ten organs using images from the Medical Segmentation Decathlon (MSD) [18,19].

Overall, the main contributions of our work are:

- We abandoned the encoder–decoder structure and considered the design of the segmentation network holistically from the dynamical systems perspective.
- We introduced RK methods into the segmentation network and inventively used various dimensions within one time step of RK methods.
- We proposed a novel segmentation network architecture called RKSeg.

### 1.1. Related Work

#### 1.1.1. Segmentation Networks

First, the classification models AlexNet [20], VGG net [21], and GoogLeNet [22], were adapted into fully convolutional networks (FCNs) [7] for semantic segmentation. Specifically, the fully connected layers were cast into convolutional layers. Moreover, up-sampling layers were added at the end of networks for pixel-wise prediction. Based on the single-stream FCN, they combine the predictions from multiple layers to improve performance.

Afterward, Reference [8] modifies and extends FCN. The backbone is no longer an existing classification model but a new design. Furthermore, the up-sampling part of FCN is expanded in order to transform FCN into a U-shaped architecture, the so-called U-Nets. U-Nets focus on biomedical image segmentation. Next, densely connected convolutional networks (DenseNets) [23] are merged into U-Nets. As a result, FC-DenseNets [24] are proposed. Based on U-Nets, UNet++ [9] is proposed as a nested U-Net architecture with deep supervision. Subsequently, UNet 3+ [10] surpassed U-Net and UNet++ on two organ datasets. Then, nnU-Nets [25] optimized U-Nets and become the state-of-the-art segmentation models. nnU-Nets proved their generalizability on the ten organ datasets of MSD. U-shaped models are dominant in medical image segmentation.

On the other side, DeepLab [26] combines the responses at the final layer of deep convolutional neural networks with a fully connected conditional random field (CRF). Then, DeepLabv2 [27] applies the atrous convolution, i.e., convolution with upsampled filters, to DeepLab. Next, DeepLabv3 [28] augments the effects of atrous convolutions and abandons CRF. DeepLabv3+ [29] improves DeepLabv3 with the encoder–decoder structure.

The state-of-the-art segmentation networks consider the down-sampling part originating from the classification network as the backbone or encoder and the up-sampling part as the decoder. These perspectives divide the semantic segmentation process into two parts and connect them via skip connections. However, these designs are experimental. To be specific, FCNs use two skip connections of small scales to improve precision. U-Net connects downstream and upstream paths in pairs by scale. UNet++ overlays U-Nets of various depths and densely connects them at each scale. UNet3+ introduces full-scale skip connections. DeepLabv3+ uses a skip connection at a medium scale. In general, they have no clear mathematical explanation for whether a node or a skip connection is necessary.

#### 1.1.2. RKCNNs

RK methods are divided into explicit methods and implicit methods. The explicit RK methods are easy to implement using a neural network to approximate ODE. However, the equations of implicit RK methods are too complicated to compute directly. In an ordinary way, Newton iterations are used to approximate the implicit RK equation. However, RKCNNs approximate the RK equations using neural networks no matter whether they are explicit or implicit. Furthermore, the coefficients of RK methods are learned through training but not specified as in other models. More details of RK methods and RKCNNs are introduced below.

A neural network stands for a time-dependent dynamical system. The system state *y* is a function of time *t*. Moreover, the rate of change of *y* is described by ODE [30]:

(1) $$\frac{dy}{dt}=f\left(t,\;y(t)\right),\;y\left(t_{0}\right)=y_{0},$$

where $y_{0}$ is the initial value. The RK methods use the ODE to approximate the system state after a time step. This approximation can be performed step by step. The (n+1)th time step of RK methods is written as below [31]:

(2) $$y_{n+1}=y_{n}+h\sum_{i=1}^{s}b_{i}z_{i},\;t_{n+1}=t_{n}+h,\;n\geq 0,$$

where

(3) $$z_{i}=f\left(t_{n}+c_{i}h,\;y_{n}+h\sum_{j=1}^{s}a_{ij}z_{j}\right),\;1\leq i\leq s.$$

The system state at time $t_{n+1}$ is $y(t_{n+1})$. In Equation (2), $y_{n+1}$ is an approximation of $y(t_{n+1})$; *h* is the size of the (n+1)th time step; $h\sum_{i=1}^{s}b_{i}z_{i}$ is the increment of *y* after *h*. The slope $z_{i}$ of the *i*th stage is computed using Equation (3). For *s*-stage RK methods, the estimated slope is a weighted average of all *s* slopes. i.e., $\sum_{i=1}^{s}b_{i}z_{i}$. $a_{ij}$, $b_{i}$, and $c_{i}$ are the coefficients of RK methods and co-decide the accuracy of the approximation.

RKCNNs are constructed based on the above equations. RKCNN contains three components: the pre-processor, the post-processor, and the periods between the former two. The raw images are processed by the pre-processor, which outputs an initial value to subsequent periods. The last period outputs to the post-processor. Next, the classifier makes predictions. For different datasets, the number of periods is various. If there are multiple periods, there are transition layers between different periods. Moreover, the transition layers reduce the dimension. Large-scale or complex images can be processed at multiple scales to improve performance. RKCNNs on the MNIST dataset have only one period, since all in MNIST are handwritten 0 to 9 gray-scale images of 28×28 pixels. Nevertheless, RKCNNs on the SVHN [32] and CIFAR [33] datasets are three-period, since all in both datasets are complex color images of 32×32 pixels. In addition, each period could be divided into multiple time steps.

If $hb_{i}z_{i}$ is denoted by $e_{i}$, Equation (2) is rewritten as [16]:

(4) $$y_{n+1}=y_{n}+\sum_{i=1}^{s}e_{i}.$$

In RKCNNs, the convolutional subnetwork for every time step is constructed based on Equation (4). There are three different architectures of RKCNN, and they are distinguished by the suffixes -E, -I, and -R. The difference among these architectures is how to approximate $e_{i}$.

In RKCNN-E, $e_{i}$ is approximated by a network $E_{i}$ as follows [16]:

(5) $$e_{i}=E_{i}\left(y_{n},\;e_{1},\;\ldots ,\;e_{i-1}\right).$$

The parameter of this network is a function of $t_{n}$, $a_{ij}$, $b_{i}$, and $c_{i}$. i.e., $t_{n}$, $a_{ij}$, $b_{i}$, and $c_{i}$ are learned through training rather than being specified [16].

RKCNN-I and RKCNN-R use Equation (5) to approximate $x_{i}$, which is the initial value of $e_{i}$[16]. i.e., $x_{i}$ is written as below:

(6) $$x_{i}=X_{i}\left(y_{n},\;x_{1},\;\ldots ,\;x_{i-1}\right).$$

In RKCNN-I, a network as shown below approximates $e_{i}$ using $x_{i}$ [16]:

(7) $$e_{i}=I_{i}\left(y_{n},\;e_{1},\;\ldots ,\;e_{i-1},\;x_{i+1},\;\ldots ,\;x_{s}\right).$$

In RKCNN-R, the network for approximating $e_{i}$ is slightly different from Equation (7). It is written as follows [16]:

(8) $$e_{i}=R_{i}\left(y_{n},\;x_{1},\;\ldots ,\;x_{i-1},\;x_{i+1},\;\ldots ,\;x_{s}\right).$$

We introduce RKCNNs into semantic segmentation. Table A1 describes all of the above mathematical symbols.

## 2. Materials and Methods

### 2.1. Architecture of RKSegs

#### 2.1.1. RKCNN-Based FCN

**Figure 1:**
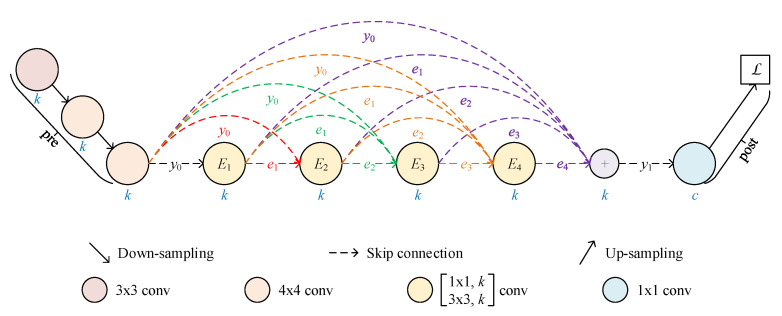
FCN with a 4-stage RKCNN-E as the backbone. $y_{0}$ is the initial state, while $y_{1}$ is the system state after one time step. $E_{i}$ is the convolutional subnetwork for the *i*th stage described in Equation (5), where 1≤i≤4. $e_{i}$ is the weighted increment of the *i*th stage. According to the RK method, $y_{1}$ is the sum of $y_{0}$ and the weighted average of the increments. *k* is the number of output channels per subnetwork. *c* is the number of classes.

We make the prototype of RKSegs based on single-stream FCNs. At first, RKCNNs are classification networks. Therefore, we can adapt them to FCNs. Since the MNIST dataset and many medical image datasets are grey-scale maps, we choose the RKCNNs for MNIST as the backbone of FCNs. Specifically, down-samplings in the pre-processor are the same as in the original models. The pooling layer before the full connection is removed. The full connection in the post-processor is changed to 1×1 convolution for pixel-wise prediction. In addition, up-sampling is appended at the end of the network. Since truncation errors can accumulate over multiple time steps [34], only one time step is used like in the original models, i.e., n=0. Then, we get RKCNN-based FCNs as the prototype of RKSegs. An example is shown in Figure 1.

#### 2.1.2. From FCN to RKSeg

The prototype of RKSeg is FCN, which uses the RKCNNs for the MNIST dataset as the backbone. The image size of the MNIST dataset is 28×28 pixels. However, the medical images are much larger than this size. Hence, we must improve the model. Additionally, the major computations in the prototype are on the same scale. Nevertheless, multiple scales can bring benefits to segmentation. Thus, we must consider the scheme to reduce the dimension.

We remove down-sampling from the pre-processor and add down-sampling bef in order to preserve more multi-scale information. If RKCNN-E is used as the backbone, based on Equations (4) and (5), the model is described as follows:

(9) $$y_{1}=y_{0}+\sum_{i=1}^{s}U\left(e_{i}\right),$$

where

(10) $$e_{i}=E_{i}\left(D\left(y_{0}\right),\;D\left(e_{1}\right),\;\ldots ,\;D\left(e_{i-1}\right)\right).$$

**Figure 2:**
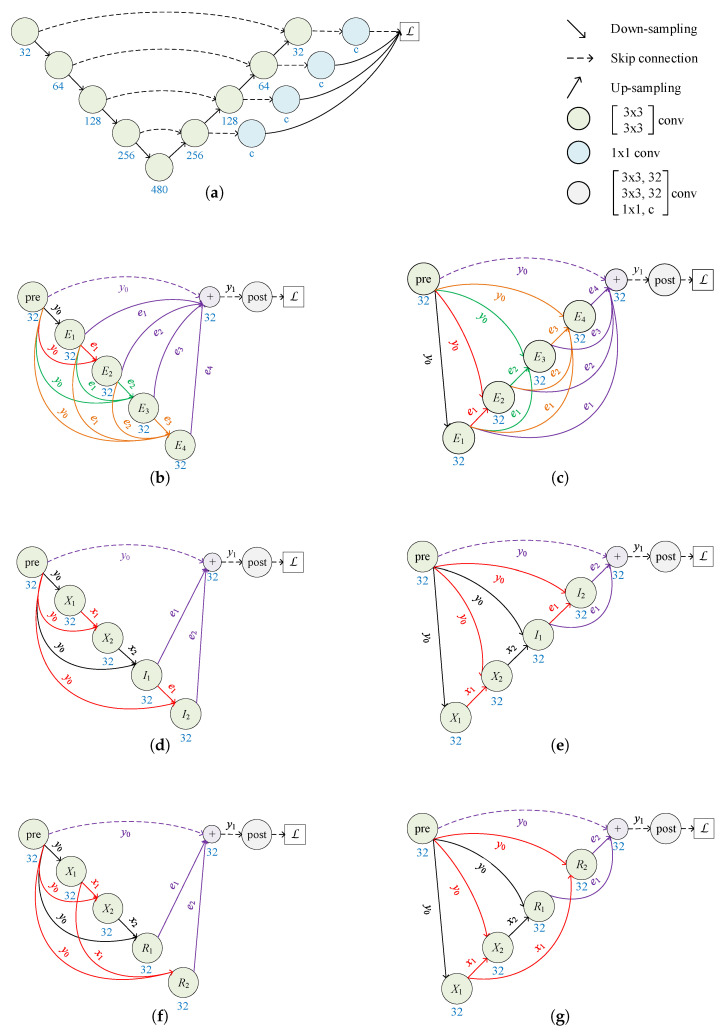
Comparison of nnU-Net and proposed RKSegs. The number under each node denotes the number of output channels. *c* is the number of classes. (**a**) nnU-Net. (**b**) RKSeg-L based on RKCNN-E. $E_{i}$ is the subnetwork described in Equation (10). (**c**) RKSeg-R based on RKCNN-E. $E_{i}$ is the subnetwork described in Equation (14). (**d**) RKSeg-L based on RKCNN-I. $X_{i}$ is the subnetwork described in Equation (11). $I_{i}$ is the subnetwork described in Equation (12). (**e**) RKSeg-R based on RKCNN-I. $X_{i}$ is the subnetwork described in Equation (15). $I_{i}$ is the subnetwork described in Equation (16). (**f**) RKSeg-L based on RKCNN-R. $X_{i}$ is the subnetwork described in Equation (11). $R_{i}$ is the subnetwork described in Equation (13). (**g**) RKSeg-R based on RKCNN-R. $X_{i}$ is the subnetwork described in Equation (15). $R_{i}$ is the subnetwork described in Equation (17).

In Equation (9), U(·) is a function for up-sampling $e_{i}$ as the same scale as $y_{0}$. In Equation (10), D(·) is a function for down-sampling input as the same scale as $e_{i}$. The resulting model is shown in Figure 2b. It is referred to as RKSeg-L, since the core of the network is on the left.

In consideration of the superiority of nnU-Nets on medical image segmentation, we use nnU-Nets for reference to adapt RKSegs. In nnU-Nets, the subnetwork in each node is $\left[\begin{array}{c} \begin{array}{c} 3\times 3,\;m\\ 3\times 3,\;m \end{array} \end{array}\right]$ convolutional layers, where *m* is the number of output channels. *m* gradually doubles as the scale gets smaller until 480. In RKSegs, we use $\left[\begin{array}{c} \begin{array}{c} 3\times 3,\;k\\ 3\times 3,\;k \end{array} \end{array}\right]$ convolutional layers, where *k* does not change but remains the same as the initial number. Like the last node of nnU-Nets, the post-processor of RKSegs is $\left[\begin{array}{c} \begin{array}{c} 3\times 3,\;k\\ 3\times 3,\;k\\ 1\times 1,\;c \end{array} \end{array}\right]$ convolutional layers, where *c* is the number of classes.

In addition, except for the first stage, every stage in RKSegs has multi-scale input. Therefore, the convolutional down-sampling in nnU-Nets is not applicable for RKSegs. We adopt MaxPool for down-sampling and interpolation for up-sampling in RKSegs. Although deep supervision is helpful to UNet++, UNet 3+, and nnU-Net, we do not use it in RKSegs, since it cannot be explained from the dynamical systems view.

Similarly, to construct RKSeg-L with RKCNN-I or RKCNN-R as the backbone, we rewrite Equation (6) as below:

(11) $$x_{i}=X_{i}\left(D\left(y_{0}\right),\;D\left(x_{1}\right),\;\ldots ,\;D\left(x_{i-1}\right)\right).$$

For RKSeg-L based on RKCNN-I, we rewrite Equation (7) as below:

(12) $$e_{i}=I_{i}\left(D\left(y_{0}\right),\;D\left(e_{1}\right),\;\ldots ,\;D\left(e_{i-1}\right),\;D\left(x_{i+1}\right),\;\ldots ,\;D\left(x_{s}\right)\right).$$

For RKSeg-L based on RKCNN-R, we rewrite Equation (8) as below:

(13) $$e_{i}=R_{i}\left(D\left(y_{0}\right),\;D\left(x_{1}\right),\;\ldots ,\;D\left(x_{i-1}\right),\;D\left(x_{i+1}\right),\;\ldots ,\;D\left(x_{s}\right)\right).$$

The resulting models are shown in Figure 2d,f.

#### 2.1.3. More Variants

In RKSeg-L, the computations of stages are from large scale to small scale. This sequence can be reversed. Therefore, we down-sample the initial state $y_{0}$ and then up-sample after each stage. In other words, for RKSeg based on RKCNN-E, Equation (5) is rewritten as below:

(14) $$e_{i}=E_{i}\left(D\left(y_{0}\right),\;V\left(e_{1}\right),\;\ldots ,\;V\left(e_{i-1}\right)\right).$$

In Equation (14), V(·) is a function for up-sampling input as the same scale as $e_{i}$. This type of RKSeg is shown in Figure 2c. It is referred to as RKSeg-R, since the core of the network is on the right.

Similarly, to construct RKSeg-R with RKCNN-I or RKCNN-R as the backbone, we rewrite Equation (6) as below:

(15) $$x_{i}=X_{i}\left(D\left(y_{0}\right),\;V\left(x_{1}\right),\;\ldots ,\;V\left(x_{i-1}\right)\right).$$

For RKSeg-R based on RKCNN-I, we rewrite Equation (7) as below:

(16) $$e_{i}=I_{i}\left(D\left(y_{0}\right),\;V\left(e_{1}\right),\;\ldots ,\;V\left(e_{i-1}\right),\;V\left(x_{i+1}\right),\;\ldots ,\;V\left(x_{s}\right)\right).$$

For RKSeg-R based on RKCNN-R, we rewrite Equation (8) as below:

(17) $$e_{i}=R_{i}\left(D\left(y_{0}\right),\;V\left(x_{1}\right),\;\ldots ,\;V\left(x_{i-1}\right),\;V\left(x_{i+1}\right),\;\ldots ,\;V\left(x_{s}\right)\right).$$

The resulting models are shown in Figure 2e,g.

According to the comparison in Figure 2, the number of nodes in RKSegs is almost half that in nnU-Nets with the same down-sampling depth. Moreover, the number of feature maps is reduced remarkably. Most importantly, nodes and skip connections of RKSegs are justified in the RK method.

### 2.2. Experiments

We evaluate RKSegs and state-of-the-art segmentation networks on the MSD dataset. The MSD tests the generalisability of algorithms when applied to 10 different semantic segmentation tasks. It involves ten organs: brain, heart, liver, hippocampus, prostate, lung, pancreas, hepatic vessel, spleen, and colon. Some medical images in MSD are MRI scans, and others are CT scans.

Owing to the superiority of nnU-Nets on MSD, we evaluate UNet++, UNet 3+, and RKSeg following the configuration of nnU-Nets, i.e., they have the same initial number of feature maps, depth of down-sampling, convolutions, and loss function as nnU-Nets on each organ dataset. However, DeepLabv3+ and FC-DenseNet do not follow these configurations since they are very different from UNets. Moreover, nnU-Net, UNet++, and UNet 3+ adopt deep supervision, while RKSeg, DeepLabv3+, and FC-DenseNet do not use deep supervision. For efficiency, MobileNetV2 [35] is used as the backbone for DeepLabv3+. FC-DenseNet56 is evaluated. All of the evaluated models use 2D convolutions.

We implement RKSegs within the nnU-Net framework, which is written using Pytorch. The code of RKSegs is available at https://github.com/ZhuMai/RKSeg (accessed on 26 March 2023). It can be integrated into the nnU-Net framework to train.

The nnU-Net framework creates a five-fold cross-validation using all of the available training cases in MSD. We do not carry out cross-validation for RKSegs but entirely follow the configuration and training hyperparameters of nnU-Nets. For example, stochastic gradient descent with an initial learning rate of 0.01 and a Nesterov momentum of 0.99 is used. Moreover, RKSegs are trained in the same batch sizes as nnU-Nets.

We choose the first fold split by nnU-Net, i.e., fold 0, to evaluate all the competitive models since MSD does not release the ground truth of the testing cases. All of the evaluated models are trained from scratch for 150 epochs on GeForce RTX 3080 GPU. The training is carried out three times. The testing cases are only used to evaluate the inference time. All the data are pre-processed by the nnU-Net framework. Details of the data used in our experiments are listed in Table 1.

We first compare RKSegs with different backbones, i.e., RKCNN-E, RKCNN-I, and RKCNN-R. The stages are alternately updated in RKCNN-I and RKCNN-R so it has at least two stages [16]. Moreover, the number of subnetworks in a time step must be even. In the configuration of nnU-Nets, the depth of down-sampling is even only on the heart and prostate datasets. Therefore, we only compare RKSegs with different backbones on the heart and prostate datasets. Next, we compare RKSegs with state-of-the-art models on all ten organ datasets of MSD.

## 3. Results

### 3.1. Comparison of Backbones

Dice similarity coefficients (DSCs) are evaluated on the validation sets of MSD. The number of parameters and DSCs are listed in Table 2. For the shown models of the prostate, the mean DSC of the two segmentation targets is the highest among the three runs.

According to the experimental results, RKSegs based on RKCNN-E get higher DSCs than the others. Furthermore, they have no limitation on the depth of down-sampling, so they can be used on more datasets. As a result, we consider that RKCNN-E is more suitable as the backbone of RKSegs.

### 3.2. Compared to State-of-the-Art Models

We compare RKSegs based on RKCNN-E with state-of-the-art models. If there are multiple segmentation targets on an organ dataset, we choose the model with the highest mean of all targets across the three runs. The experimental data are shown in Table 3.

Furthermore, we evaluate the inference time of the competitive models on testing cases of MSD. The results are shown in Table 4.

**Figure 3:**
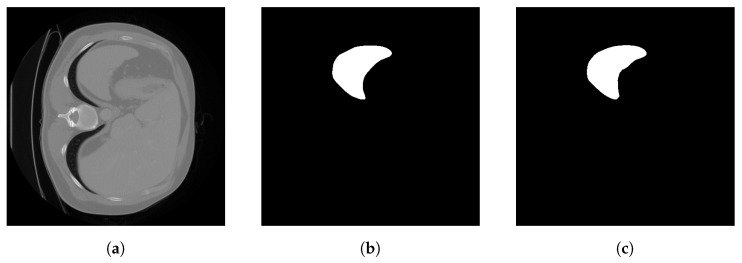
Demonstration of the segmentation result of RKSeg-L on the spleen dataset. (**a**) Raw image. (**b**) Label. (**c**) Segmentation of RKSeg-L.

A segmentation result of RKSeg-L on the spleen dataset is shown in Figure 3. RKSeg-L can even segment more curved details on the right side of the spleen. Its segmentation result is more like the raw image than the label.

## 4. Discussion

We construct RKSegs based on RKCNNs. If RKCNN-I or RKCNN-R has the same number of nodes as RKCNN-E, then it has half the stages of RKCNN-E, because it updates each stage alternately [16]. Therefore, the number of skipped connections from the stages to the addition in RKCNN-I or RKCNN-R is only half of that in RKCNN-E. Hence, RKSegs based on RKCNN-E have more information from different stages at different scales. Multi-scale information can bring benefits to segmentation. As expected, RKSegs based on RKCNN-E achieve higher DSCs than corresponding RKSegs based on RKCNN-I or RKCNN-R. As a result, RKSegs based on RKCNN-E are chosen for comparison with state-of-the-art models.

According to the experimental results, our RKSegs have the fewest parameters. At the same time, RKSegs achieve the highest mean DSCs over three runs on three CT datasets, namely the lung, spleen, and colon datasets. In addition, on the brain dataset, which is an MRI dataset, RKSeg wins on a segmentation target, while UNet++ wins on the other two targets and the mean of three targets. On the other six organ datasets, RKSegs obtain competitive DSCs with only 0.85~11% of the parameters of the best models. Additionally, according to the standard deviation of three runs, RKSegs are stable. In terms of training time, RKSegs obtain the shortest time once, the second shortest time eight times, and the third shortest time once. However, the inference time of RKSegs is the shortest among all of the evaluated models.

FC-DenseNet56 achieves the highest mean DSCs over three runs on two MRI datasets, the hippocampus and prostate datasets. On the prostate dataset, FC-DenseNet56 wins on a segmentation target and the mean of two targets, while UNet++ wins on the other target. Additionally, the performance of FC-DenseNet56 is very poor on six CT datasets. nnU-Nets achieve the highest mean DSCs over three runs on one MRI dataset and three CT datasets. Nevertheless, nnU-Nets have a lot of parameters on each organ dataset. UNet++ achieves the highest mean DSC over three runs on an MRI dataset, the brain dataset. However, UNet++ has the most parameters among all of the evaluated models.

In summation, RKSegs are general and efficient on diverse organ datasets with different modalities.

For the encoder–decoder structure, skip connections are used to introduce multi-scale information in prediction. However, whether to add nodes for the decoder and where to add skip connections are up to experimentation.

Contrarily, RKSegs are constructed from the dynamical systems view. Each node and skip connection of RKSegs is justified in the RK methods. Hence, RKSegs avoid many superfluous components in other models. Computational resources are greatly saved. Experimental results show that RKSegs have much higher efficiency than competing models and generalize across diverse organ datasets. Even so, the efficiency of RKSegs still could be improved. For example, the convolutional subnetwork of each node, the depth of the network, and the training hyperparameters of RKSegs are all the same as nnU-Nets in our experiments. They can be tuned to improve the performance of RKSegs.

## 5. Conclusions

The encoder–decoder structure is a well-known structure in medical image segmentation. However, the composition of the decoder and the skip connections between the encoder and decoder are designed experimentally. Therefore, segmentation models are either inefficient or not generalizable across different organs. To remedy these deficiencies, we introduce a dynamical systems view to build segmentation models.

We propose a novel segmentation network based on RKCNNs, which use RK methods to construct networks. Our network is called RKSeg. Unlike RKCNNs, RKSegs perform down-sampling and up-sampling within a time step of the RK methods. In RKSegs, each node and skip connection is meaningful in the RK methods. According to the experiments, RKSegs based on RKCNN-E achieve superior performance on the ten organ datasets of MSD, while they have much fewer parameters than other models. Furthermore, RKSegs have a shorter inference time than competitive models on each organ dataset.

Mathematical methods bring benefits to the performance of network models. Our work may inspire new ideas about segmentation networks.

## Figures and Tables

**Table 1:** Introduction of datasets used in experiments.

Organs	Segmentation Target	Training Cases	Validation Cases	Testing Cases	Modality	Type
Brain	1: edema					
	2: non-enhancing tumor	387	97	266	Multimodal multisite MRI data	4D
	3: enhancing tumor					
Heart	left atrium	16	4	10	Mono-modal MRI	3D
Liver	1: liver	104	27	70	Portal venous phase CT	3D
	2: cancer					
Hippocampus	1: anterior	208	52	130	Mono-modal MRI	3D
	2: posterior					
Prostate	1: peripheral zone	25	7	16	Multimodal MR	4D
	2: transition zone					
Lung	cancer	50	13	32	CT	3D
Pancreas	1: pancreas	224	57	139	Portal venous phase CT	3D
	2: cancer					
Hepatic Vessel	1: vessel	242	61	140	CT	3D
	2: tumour					
Spleen	spleen	32	9	20	CT	3D
Colon	colon cancer primaries	100	26	64	CT	3D

**Table 2:** DSCs of RKSegs with different backbones. DSCs are listed sequentially according to the segmentation targets of the corresponding organs in Table 1. The mean ± std over three runs is in brackets. The unit of parameters is a million bytes. The highest DSCs are in blue.

		Heart		Prostate			
Models	Backbones	Params	Left Atrium	Params	Peripheral Zone	Transition Zone	Mean
RKSeg-L	RKCNN-E	0.28	0.9137 (0.9101 ± 0.0032)	0.28	0.6642 (0.6522 ± 0.0105)	0.8715 (0.8709 ± 0.0042)	0.7678 (0.7615 ± 0.0068)
	RKCNN-I	0.22	0.8983 (0.8937 ± 0.0033)	0.22	0.6430 (0.6392 ± 0.0071)	0.8641 (0.8604 ± 0.0027)	0.7535 (0.7498 ± 0.0040)
	RKCNN-R	0.22	0.9028 (0.9008 ±0.0022)	0.22	0.6563 (0.6510 ±0.0039)	0.8703 (0.8663 ±0.0055)	0.7633 (0.7587 ±0.0044)
RKSeg-R	RKCNN-E	0.28	0.9136 (0.9108 ± 0.0020)	0.28	0.6608 (0.6486 ± 0.0088)	0.8740 (0.8717 ± 0.0023)	0.7674 (0.7602 ± 0.0051)
	RKCNN-I	0.22	0.9117 (0.9102 ± 0.0015)	0.22	0.6553 (0.6382 ± 0.0144)	0.8694 (0.8587 ± 0.0076)	0.7624 (0.7485 ± 0.0104)
	RKCNN-R	0.22	0.9086 (0.9060 ± 0.0029)	0.22	0.6601 (0.6439 ±0.0115)	0.8733 (0.8723 ± 0.0023)	0.7667 (0.7581 ±0.0063)

**Table 3:** DSCs of competitive models on the validation sets of MSD. DSCs are listed sequentially according to the segmentation targets of the corresponding organs in Table 1. The mean ± std over three runs is in brackets. The unit of parameters is a million bytes. The training time is shown in the format of hh:mm:ss. The experimental data on ten organ datasets are divided into (**a**)~(**j**). The fewest parameters and the highest DSCs are in blue.

(a) Brain						
**Models**	**Params**	**Edema**	**Non-Enhancing Tumor**	**Enhancing Tumor**	**Mean**	**Time**
nnU-Net [25]	18.67	0.7876 (0.7869±0.0006)	0.6046 (0.6036±0.0010)	0.7527 (0.7489±0.0033)	0.7150 (0.7131±0.0015)	3:22:49
UNet++ [9]	24.00	0.7840 (0.7870 ± 0.0023)	0.6084 (0.6040±0.0031)	0.7580 (0.7578 ± 0.0004)	0.7168 (0.7163 ± 0.0006)	4:42:23
UNet 3+ [10]	11.98	0.6218 (0.6113±0.0079)	0.4137 (0.4159±0.0122)	0.4782 (0.4808±0.0137)	0.5046 (0.5027±0.0014)	22:01:57
DeepLabv3+ [29]	5.22	0.7820 (0.7785±0.0026)	0.5737 (0.5731±0.0026)	0.7235 (0.7249±0.0043)	0.6931 (0.6922±0.0007)	2:52:06
FC-DenseNet56 [24]	2.50	0.7773 (0.7780±0.0006)	0.6045 (0.6034±0.0026)	0.7556 (0.7538±0.0046)	0.7124 (0.7117±0.0009)	2:19:45
RKSeg-L (ours)	0.21	0.7865 (0.7853±0.0014)	0.6091 (0.6054±0.0037)	0.7628 (0.7565±0.0072)	0.7194 (0.7157±0.0041)	6:10:22
RKSeg-R (ours)	0.21	0.7787 (0.7818±0.0022)	0.6092 (0.6061 ± 0.0024)	0.7566 (0.7515±0.0036)	0.7148 (0.7131±0.0012)	2:42:23
**(b) Heart**						
**Models**	**Params**	**Left Atrium**				**Time**
nnU-Net [25]	29.97		0.9191 (0.9190 ± 0.0001)			2:29:16
UNet++ [9]	49.35		0.9138 (0.9136±0.0002)			2:59:31
UNet 3+ [10]	18.13		0.6555 (0.6528±0.0021)			36:17:41
DeepLabv3+ [29]	5.22		0.9000 (0.8969±0.0026)			1:15:27
FC-DenseNet56 [24]	2.49		0.9230 (0.9175±0.0039)			2:36:19
RKSeg-L (ours)	0.28		0.9137 (0.9101±0.0032)			11:19:51
RKSeg-R (ours)	0.28		0.9136 (0.9108±0.0020)			1:40:39
**(c) Liver**						
**Models**	**Params**	**Liver**	**Cancer**	**Mean**		**Time**
nnU-Net [25]	41.26	0.9586 (0.9563 ± 0.0017)	0.5662 (0.5560 ± 0.0121)	0.7624 (0.7562 ± 0.0064)		2:33:14
UNet++ [9]	86.77	0.9514 (0.9512±0.0003)	0.4861 (0.4693±0.0123)	0.7187 (0.7102±0.0062)		4:42:29
UNet 3+ [10]	25.01	0.0000 (0.0000±0.0000)	0.0000 (0.0000±0.0000)	0.0000 (0.0000±0.0000)		55:50:10
DeepLabv3+ [29]	5.22	0.9569 (0.9562±0.0005)	0.5523 (0.5455±0.0090)	0.7546 (0.7508±0.0046)		1:32:43
FC-DenseNet56 [24]	2.49	0.8545 (0.8597±0.0071)	0.2645 (0.2542±0.0074)	0.5595 (0.5570±0.0029)		2:37:57
RKSeg-L (ours)	0.35	0.9517 (0.9524±0.0013)	0.5454 (0.5257±0.0186)	0.7485 (0.7390±0.0095)		17:22:25
RKSeg-R (ours)	0.35	0.9461 (0.9458±0.0003)	0.4597 (0.4458±0.0115)	0.7029 (0.6958±0.0058)		1:57:55
**(d) Hippocampus**						
**Models**	**Params**	**Anterior**	**Posterior**	**Mean**		**Time**
nnU-Net [25]	1.93	0.8866 (0.8864±0.0001)	0.8691 (0.8689±0.0005)	0.8778 (0.8777±0.0002)		1:06:31
UNet++ [9]	2.21	0.8878 (0.8871±0.0006)	0.8698 (0.8699±0.0001)	0.8788 (0.8785±0.0002)		1:19:40
UNet 3+ [10]	2.07	0.8627 (0.8617±0.0008)	0.8372 (0.8367±0.0004)	0.8500 (0.8492±0.0005)		4:11:23
DeepLabv3+ [29]	5.22	0.8752 (0.8750±0.0004)	0.8588 (0.8582±0.0005)	0.8670 (0.8666±0.0003)		1:03:57
FC-DenseNet56 [24]	2.49	0.8932 (0.8922 ± 0.0008)	0.8751 (0.8745 ± 0.0005)	0.8841 (0.8833 ± 0.0006)		2:15:51
RKSeg-L (ours)	0.11	0.8894 (0.8886±0.0008)	0.8731 (0.8724±0.0005)	0.8813 (0.8805±0.0006)		0:49:29
RKSeg-R (ours)	0.11	0.8892 (0.8889±0.0007)	0.8736 (0.8728±0.0007)	0.8814 (0.8809±0.0007)		0:49:21
**(e) Prostate**						
**Models**	**Params**	**Peripheral Zone**	**Transition Zone**	**Mean**		**Time**
nnU-Net [25]	29.97	0.6747 (0.6685±0.0044)	0.8827 (0.8808±0.0015)	0.7787 (0.7747±0.0030)		2:34:08
UNet++ [9]	49.35	0.7129 (0.6982±0.0104)	0.8855 (0.8842 ± 0.0012)	0.7992 (0.7912±0.0056)		3:03:04
UNet 3+ [10]	18.15	0.5402 (0.5218±0.0187)	0.8381 (0.8329±0.0039)	0.6892 (0.6774±0.0113)		36:37:15
DeepLabv3+ [29]	5.22	0.6409 (0.6128±0.0206)	0.8726 (0.8666±0.0043)	0.7568 (0.7397±0.0125)		1:29:24
FC-DenseNet56 [24]	2.50	0.7149 (0.7026 ± 0.0090)	0.8875 (0.8832±0.0035)	0.8012 (0.7929 ± 0.0062)		3:14:00
RKSeg-L (ours)	0.28	0.6642 (0.6522±0.0105)	0.8715 (0.8709±0.0042)	0.7678 (0.7615±0.0068)		11:31:27
RKSeg-R (ours)	0.28	0.6608 (0.6486±0.0088)	0.8740 (0.8717±0.0023)	0.7674 (0.7602±0.0051)		1:45:07
**(f) Lung**						
**Models**	**Params**		**Cancer**			**Time**
nnU-Net [25]	41.26		0.5620 (0.5557±0.0047)			2:34:18
UNet++ [9]	86.77		0.4915 (0.4531±0.0274)			4:15:01
UNet 3+ [10]	24.99		0.0000 (0.0000±0.0000)			55:12:41
DeepLabv3+ [29]	5.22		0.5929 (0.5468±0.0326)			1:18:37
FC-DenseNet56 [24]	2.49		0.4302 (0.4173±0.0101)			2:21:04
RKSeg-L (ours)	0.35		0.5302 (0.5085±0.0163)			17:09:48
RKSeg-R (ours)	0.35		0.5961 (0.5763 ± 0.0219)			1:45:26
**(g) Pancreas**						
**Models**	**Params**	**Pancreas**	**Cancer**	**Mean**		**Time**
nnU-Net [25]	41.26	0.7418 (0.7434 ± 0.0015)	0.3644 (0.3533 ± 0.0093)	0.5531 (0.5484 ± 0.0039)		2:40:00
UNet++ [9]	86.77	0.6906 (0.6889±0.0027)	0.3393 (0.3320±0.0064)	0.5150 (0.5104±0.0044)		4:23:14
UNet 3+ [10]	25.01	0.0000 (0.0000±0.0000)	0.0000 (0.0000±0.0000)	0.0000 (0.0000±0.0000)		55:29:29
DeepLabv3+ [29]	5.22	0.6982 (0.6843±0.0110)	0.2964 (0.2821±0.0102)	0.4973 (0.4832±0.0102)		1:24:22
FC-DenseNet56 [24]	2.49	0.3575 (0.3332±0.0207)	0.2067 (0.1941±0.0104)	0.2821 (0.2637±0.0156)		2:25:15
RKSeg-L (ours)	0.35	0.7090 (0.7084±0.0034)	0.3395 (0.3264±0.0104)	0.5242 (0.5174±0.0063)		17:13:50
RKSeg-R (ours)	0.35	0.6798 (0.6802±0.0016)	0.3193 (0.3007±0.0179)	0.4995 (0.4904±0.0094)		1:49:43
**(h) Hepatic Vessel**						
**Models**	**Params**	**Vessel**	**Tumour**	**Mean**		**Time**
nnU-Net [25]	41.26	0.6631 (0.6603 ± 0.0026)	0.6548 (0.6500 ± 0.0035)	0.6590 (0.6552 ± 0.0027)		2:38:24
UNet++ [9]	86.77	0.6522 (0.6512±0.0036)	0.6419 (0.6322±0.0078)	0.6471 (0.6417±0.0038)		4:20:34
UNet 3+ [10]	25.01	0.1120 (0.0921±0.0221)	0.2638 (0.2646±0.0043)	0.1879 (0.1784±0.0132)		55:24:44
DeepLabv3+ [29]	5.22	0.6392 (0.6343±0.0041)	0.6473 (0.6358±0.0082)	0.6432 (0.6350±0.0060)		1:23:00
FC-DenseNet56 [24]	2.49	0.5923 (0.5864±0.0043)	0.3963 (0.3599±0.0270)	0.4943 (0.4731±0.0156)		2:29:33
RKSeg-L (ours)	0.35	0.6522 (0.6507±0.0019)	0.6372 (0.6328±0.0033)	0.6447 (0.6417±0.0021)		17:13:56
RKSeg-R (ours)	0.35	0.6475 (0.6465±0.0009)	0.5971 (0.5706±0.0195)	0.6223 (0.6085±0.0101)		1:49:12
**(i) Spleen**						
**Models**	**Params**		**Spleen**			**Time**
nnU-Net [25]	41.26		0.9082 (0.9059±0.0017)			2:27:17
UNet++ [9]	86.77		0.9101 (0.8960±0.0119)			3:47:19
UNet 3+ [10]	24.99		0.5711 (0.5561±0.0153)			54:49:08
DeepLabv3+ [29]	5.22		0.9228 (0.9158±0.0062)			1:13:43
FC-DenseNet56 [24]	2.49		0.5632 (0.4762±0.0774)			2:07:48
RKSeg-L (ours)	0.35		0.9222 (0.9151±0.0055)			17:09:42
RKSeg-R (ours)	0.35		0.9171 (0.9165 ± 0.0006)			1:41:05
**(j) Colon**						
**Models**	**Params**		**Colon Cancer Primaries**			**Time**
nnU-Net [25]	41.26		0.2718 (0.2434±0.0202)			2:32:09
UNet++ [9]	86.77		0.2088 (0.1844±0.0184)			3:01:07
UNet 3+ [10]	24.99		0.0000 (0.0000±0.0000)			36:55:21
DeepLabv3+ [29]	5.22		0.2082 (0.1929±0.0169)			1:17:03
FC-DenseNet56 [24]	2.49		0.1496 (0.1022±0.0336)			1:38:18
RKSeg-L (ours)	0.35		0.2960 (0.2857 ± 0.0122)			17:07:55
RKSeg-R (ours)	0.35		0.2311 (0.2248±0.0066)			1:43:32

**Table 4:** Average inference time per test case in MSD. The unit of time is second. The shortest time is in blue.

Models	Brain	Heart	Liver	Hippocampus	Prostate	Lung	Pancreas	Hepatic Vessel	Spleen	Colon
nnU-Net [25]	2.93	5.60	42.25	0.42	0.93	24.18	8.82	6.16	8.07	7.53
UNet++ [9]	5.47	8.35	135.64	0.58	1.55	108.13	40.60	27.21	36.84	35.43
UNet 3+ [10]	5.62	8.85	136.35	0.64	1.75	111.54	40.41	27.77	37.60	35.21
DeepLabv3+ [29]	3.61	4.83	43.39	0.81	0.91	22.24	8.28	5.71	7.42	7.31
FC-DenseNet56 [24]	4.60	6.10	82.42	0.98	1.21	60.08	23.37	15.85	21.27	20.31
RKSeg-L (ours)	2.06	3.48	37.55	0.31	0.75	18.63	6.49	4.56	5.89	5.55
RKSeg-R (ours)	2.21	3.46	37.00	0.33	0.71	20.34	7.08	4.93	6.34	5.90

## Data Availability

The datasets analyzed during the current study are available on the MSD website, http://medicaldecathlon.com/ (accessed on 26 March 2023).

## Abbreviations

The following abbreviations are used in this manuscript:

MNIST	Modified National Institute of Standards and Technology
MSD	Medical Segmentation Decathlon
RK	Runge–Kutta
ODE	Ordinary Differential Equation
MRI	Magnetic Resonance Imaging
CT	Computerized Tomography

## Appendix A

An introductions to the math symbols mentioned in the main text in summarized in Table A1.

**Table A1:** Introductions to the math symbols in the main text.

Math Symbols	Introduction
*t*	A scalar representing time.
*y*	A vector representing the state of a dynamical system.
y(t)	*y* is a function of *t*.
$\frac{dy}{dt}$	The rate of change of the system state.
$t_{n}$	The *n*th moment.
$t_{0}$	The initial time.
$y(t_{n})$	The system state at $t_{n}$.
$y_{n}$	An approximation of $y(t_{n})$.
$y_{0}$	The initial state of the system.
*h*	The size of the time step from $t_{n}$ to $t_{n+1}$.
*s*	The stages of RK methods.
$z_{i}$	The slope in the *i*th stage.
$a_{ij}$	A coefficient of RK methods. It indicates the dependence of the stages on the derivatives found at other stages.
$b_{i}$	A coefficient of RK methods. All $b_{i}$ are quadrature weights, showing how the final result depends on the derivatives computed at the various stages.
$c_{i}$	A coefficient of RK methods. It indicates the position of the stage value within the time step.
$\sum_{i=1}^{s}b_{i}z_{i}$	The weighted average of $z_{i}$ as the estimated slope.
$h\sum_{i=1}^{s}b_{i}z_{i}$	The increment of the system state after the duration *h*.
$hb_{i}z_{i}$	The weighted increment of the *i*th stage.
$e_{i}$	It stands for $hb_{i}z_{i}$.
$x_{i}$	The initial value of $e_{i}$ in RKCNN-I and RKCNN-R.
$E_{i}$	The convolutional subnetwork to get $e_{i}$ in RKCNN-E.
$X_{i}$	The convolutional subnetwork to get $x_{i}$ in RKCNN-I or RKCNN-R.
$I_{i}$	The convolutional subnetwork to get $e_{i}$ in RKCNN-I.
$R_{i}$	The convolutional subnetwork to get $e_{i}$ in RKCNN-R.
*m*	The number of convolution filters. It is variable.
*k*	The number of convolution filters. It is constant.
*c*	The number of classes.
